# Erie County Medical Society

**Published:** 1881-06

**Authors:** 


### ERIE COUNTY MEDICAL SOCIETY.

### JUNE MEETING.

THE June meeting of the Erie County Medical Society will be held at the Medical College in this city, Tuesday, June 14, commencing its session at 10 A. M. In addition to the usual business, which usually occupies the attention of the Society, the enforcement of the new medical law, which has been in charge of the Board of Censors, will come up for consideration. We feel that prompt action should be taken in a matter which concerns so deeply the profession and the public. Recent events have only magnified the importance of this subject, and the Society, in its corporate capacity, cannot with any consistency delay action, with a view to the enforcement of this law.

